# Efficacy and Safety of Glutathione Supplementation in Type 2 Diabetes & Diabetes Complications

**DOI:** 10.3390/nu18132132

**Published:** 2026-07-01

**Authors:** Stefanie Au, John Dawi, Scarlet Affa, Yura Misakyan, Edgar Gonzalez, Abraham Chorbajian, Mary Hammi, Priyanka Dave, Kyla Qumsieh, Vishwanath Venketaraman

**Affiliations:** 1 College of Osteopathic Medicine of the Pacific, Western University of Health Sciences, Pomona, CA 91766, USAjohn.dawi@westernu.edu (J.D.); yura.misakyan@westernu.edu (Y.M.); edgar.gonzalez@westernu.edu (E.G.); abraham.chorbajian@westernu.edu (A.C.); priyanka.dave@westernu.edu (P.D.); kyla.qumsieh@westernu.edu (K.Q.); 2 Department of Biochemistry and Chemistry, The College, UCLA, 405 Hilgard Avenue, Los Angeles, CA 90095, USA; scarleta@ucla.edu

**Keywords:** glutathione, oxidative stress, type 2 diabetes mellitus (T2DM), N-acetylcysteine (NAC)

## Abstract

Glutathione (GSH), the most abundant intracellular antioxidant, plays a central role in maintaining redox homeostasis, regulating immune responses, and protecting cellular integrity. In chronic diseases such as type 2 diabetes mellitus (T2DM), GSH deficiency is a consistent hallmark, contributing to oxidative stress, mitochondrial dysfunction, inflammation, and progressive organ damage. This review critically examines the efficacy and safety of GSH supplementation and precursor strategies, synthesizing evidence across mechanistic studies, clinical trials, and translational research. In T2DM, GSH augmentation has been linked to improved insulin sensitivity, reduced oxidative damage, and better microvascular outcomes, although findings remain preliminary and heterogeneous. Safety profiles across populations are highly favorable, with gastrointestinal discomfort being the most reported adverse effect and serious toxicities rare. Importantly, both acute and chronic studies reinforce the compatibility of GSH and its precursors with standard antiretroviral and antidiabetic therapies. Despite this encouraging data, significant research gaps remain. Standardization of biomarkers, dose–response mapping, and long-term outcomes are urgently needed to move from proof-of-concept to clinical trials. Future directions include integrating mechanistic endpoints such as mitochondrial function and multi-omic profiling, exploring targeted delivery systems, and embedding implementation science to ensure real-world feasibility and equity. Collectively, the emerging evidence supports GSH-centered strategies as promising adjuncts for oxidative stress-driven chronic disease. Rigorous, well-designed trials are now required to define their definitive role in clinical care.

## 1. Introduction

Glutathione (GSH), a tripeptide composed of glutamate, cysteine, and glycine, is the central low-molecular-weight thiol in mammalian cells and the dominant intracellular antioxidant. Concentrations can reach up to 10 mmol/L in hepatocytes, underscoring its indispensable role in redox regulation. Its biosynthesis involves two ATP-dependent enzymatic steps, catalyzed first by glutamate cysteine ligase, the rate-limiting enzyme, and then by glutathione synthetase. The availability of cysteine is the most significant determinant of intracellular GSH levels, making it vulnerable to nutritional and metabolic perturbations. GSH functions by maintaining thiol groups in proteins in a reduced state, detoxifying electrophiles via glutathione S-transferases, and serving as a cofactor for glutathione peroxidases that neutralize hydrogen peroxide and lipid peroxides. Recycling of oxidized glutathione (GSSG) back to GSH by glutathione reductase consumes NADPH, thereby linking GSH cycling directly to cellular energy and mitochondrial metabolism [1].

The pathophysiological importance of glutathione is evident in type 2 diabetes mellitus (T2DM), a disease marked by persistent hyperglycemia, chronic oxidative stress, and accelerated vascular injury. Excess glucose drives reactive oxygen species (ROS) generation through mitochondrial electron leakage, activation of the polyol pathway, and protein kinase C signaling, overwhelming the cellular antioxidant network [1]. Thus, GSH supplementation and precursors such as N-acetylcysteine (NAC) are a promising solution in the management of T2DM, a simple intervention that has the potential to correct GSH deficiency, restore mitochondrial function, and mitigate oxidative injury (Table 1).

## 2. Methodology

### 2.1. Search Strategy

A structured literature search was performed in PubMed, Medline, and Google Scholar databases to identify relevant studies examining GSH supplementation, redox balance, and oxidative stress in the context of type 2 diabetes mellitus (T2DM). The search was conducted up to December 2025 using combinations of the following keywords and Boolean operators: “glutathione” AND (“type 2 diabetes” OR “T2DM” OR “insulin resistance”) AND (“oxidative stress” OR “mitochondrial dysfunction” OR “redox”) AND (“N-acetylcysteine” OR “GlyNAC” OR “GSH”). The following filters were applied to the search terms when applicable: studies between 2015 and 2025, involves glutathione supplementation, published in the English language or had a translation available, and used any variation of glutathione (including acetylcysteine and GlyNAC). Articles were initially screen by titles and abstracts before full-text review. Reference lists from major publications were manually reviewed to ensure completeness and capture of related cohort data. A total of 96 articles were identified through initial screening.

### 2.2. Inclusion Criteria

Final inclusion criteria comprised peer-reviewed original research articles, clinical trials, and mechanistic or translational studies involving human participants, animal models, or in vitro systems that evaluated GSH pathways, redox balance, mitochondrial function, or supplementation strategies in T2DM. Studies were required to report measurable biochemical, metabolic, or clinical outcomes related to oxidative stress or antioxidant capacity in order to be included. Articles on GSH in related diseases such as aging and metabolic syndrome were also included in the study.

### 2.3. Exclusion Criteria

Exclusion criteria included non-English publications, case reports, conference abstracts, editorials, narrative opinions, and studies lacking quantitative redox, metabolic, or mitochondrial outcome measures. Studies focusing exclusively on unrelated infectious, oncologic, or immunologic outcomes without assessment of oxidative pathways were also excluded. After title and abstract review, 68 articles were excluded due to irrelevance, duplication, lack of translational outcome parameters, or lack of adequate and direct discussion of GSH supplementation in T2DM.

### 2.4. Analytical Approach

The remaining 28 studies were synthesized in the review. Data was extracted by nine independent reviewers to summarize and analyze literature findings to incorporate various study designs, interventions, and outcomes. A narrative synthesis approach was employed due to the substantial heterogeneity among included studies with respect to study design, participant populations, intervention strategies, outcome measures, and analytical methods. A narrative framework was therefore selected to integrate mechanistic, translational, and clinical evidence, allowing for contextual interpretation of findings and identification of emerging themes across diverse experimental models.

## 3. Background

### 3.1. Glutathione Supplementation in T2DM’s Mitochondrial Redox Pathways

The mechanistic premise is straightforward: cysteine supplies the rate-limiting substrate for γ-glutamylcysteine formation, while glycine ensures completion of the tripeptide, thereby maximizing the capacity of glutamate-cysteine ligase and GSH synthetase. Clinical signals across mitochondrial and metabolic endpoints argue that redox repletion influences upstream bioenergetics as well as downstream glucose handling [2,3]. Safety and tolerability profiles were acceptable, a prerequisite for longer and broader trials in heterogeneous T2DM cohorts. Although sample sizes were modest, effect directions were biologically coherent and consistent with prior metabolic physiology. These findings justify larger, adequately powered studies targeting cardiometabolic outcomes beyond biochemical markers alone. Collectively, GlyNAC (glycine plus N-acetylcysteine) offers a translational bridge from mechanism to metabolism in T2DM care.

**Figure 1:**
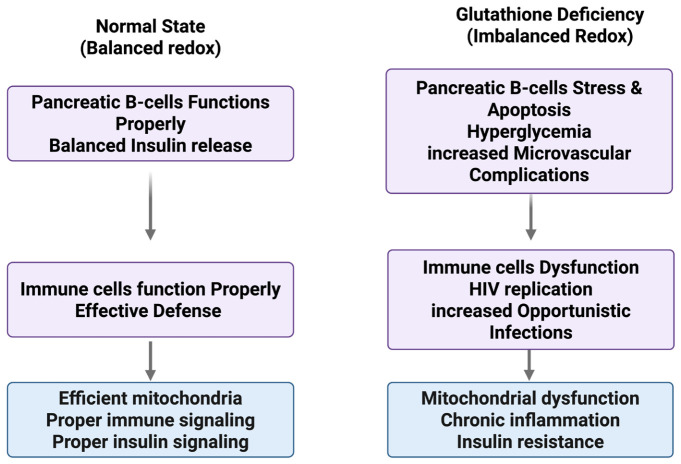
Pathophysiological Roles of Glutathione Deficiency in T2DM.

**Figure 2:**
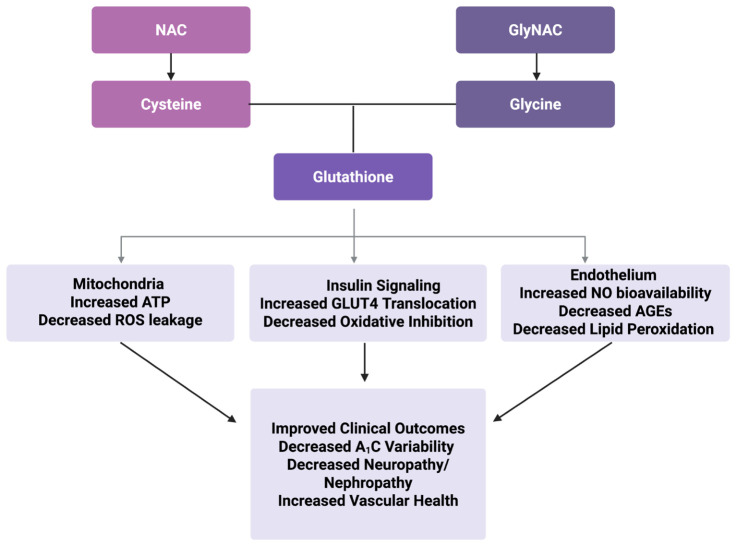
Mechanistic Targets of GSH Supplementation in Type 2 Diabetes. NAC—N-acetylcysteine; GlyNAC—Glycine plus N-acetylcysteine.

Metabolic studies provide evidence of GSH deficiency in T2DM. Using stable isotope tracer methodology, Sekhar demonstrated that uncontrolled diabetic patients exhibit impaired de novo GSH synthesis due to limited availability of cysteine and glycine [2]. Supplementation with these amino acids restored GSH synthesis and reduced systemic oxidative stress, confirming that precursor insufficiency is the primary bottleneck (Figure 1). This discovery reframed GSH deficiency in diabetes not as a problem of increased utilization, but of inadequate synthesis, providing a clear mechanistic rationale for targeted precursor supplementation. In a separate study trialing GlyNAC in patients with T2DM, GlyNAC was shown to improve indices of mitochondrial fuel oxidation, indicating recovery of metabolic flexibility that is frequently impaired by chronic insulin resistance. The intervention also lowered surrogate measures of oxidative stress, aligning with the biochemical expectation that restoring intracellular GSH buffers reactive species more effectively. Observed improvements in insulin resistance supported the notion that redox normalization can secondarily modulate insulin signaling pathways at the cellular level [3] (Figure 2). Improvements in mitochondrial oxidation also suggest that GSH repletion may stabilize NAD(P)H-coupled redox cycles required for oxidative phosphorylation and anaplerosis in insulin-resistant muscle and liver [3]. Insulin resistance is not solely a receptor-proximal phenomenon; mitochondrial substrate competition and incomplete fatty-acid oxidation also impose brakes on glucose flux, which GlyNAC appears to relieve indirectly. Clinical relevance stems from the breadth of endpoints—fuel oxidation, oxidative stress, and insulin sensitivity—moving the field beyond single-marker improvements. However, this intervention’s practicality in routine T2DM care must be tested in a larger trial with a placebo group. If reproduced on a larger scale with appropriate controls, GlyNAC could be positioned as a metabolic adjuvant that targets the redox underpinnings of insulin resistance in T2DM.

At the mitochondrial level, impaired Complex I NADH oxidation can degrade the glutathione redox state and propagate insulin resistance through redox-sensitive signaling nodes. A mechanistic synthesis highlights how SIRT3, a mitochondrial deacetylase, coordinates redox enzyme activity and becomes functionally compromised in insulin-resistant states. Depressed Complex I activity promotes superoxide production, increasing peroxide loads that strain GSH-dependent detoxification [4]. As GSH/GSSG ratios fall, redox-sensitive kinases and phosphatases shift insulin signaling toward resistance phenotypes. Restoring GSH thereby has the potential to stabilize mitochondrial proteins, preserve SIRT3-regulated defenses, and reduce ROS propagation. This model links thiol availability to the architecture of mitochondrial quality control and substrate handling and explains why interventions that improve GSH status can indirectly augment oxidative phosphorylation efficiency. In practice, combined strategies that improve mitochondrial function and replenish GSH may outperform either alone. Translational endpoints should therefore include mitochondrial respiration and acetylation status alongside glycemic metrics; such integrated designs would clarify causality between redox restoration and insulin sensitization.

Human genetics underscore the biological plausibility that higher intrinsic GSH synthesis protects against diabetes. Variants in the catalytic and modifier subunits of glutamate-cysteine ligase are associated with reduced T2DM risk, consistent with enhanced capacity to synthesize GSH [5]. This observation triangulates epidemiology with mechanism, anchoring GSH biology in causal inference rather than correlation alone. Genetic protection implies that lifelong advantages in thiol homeostasis confer resilience against metabolic stressors. Such findings motivate genotype-informed analyses in intervention trials to detect effect modification and support the search for small-molecule activators of GSH biosynthesis as complements to precursor strategies. Ultimately, genetics provides a mechanistic scaffold for precision deployment of glutathione-centric interventions.

ROS are increasingly recognized not only as mediators of oxidative damage but also as essential components of physiological signaling networks. Controlled levels of oxidants such as hydrogen peroxide regulate insulin receptor phosphorylation, vascular tone, and immune cell activation. On the other hand, broad or nonspecific antioxidant therapy may inadvertently suppress beneficial ROS-dependent pathways and disrupt normal redox signaling. Forman and Zhang emphasize that antioxidant strategies should focus on redox modulation rather than indiscriminate scavenging, aiming to restore balance without abolishing necessary oxidant-mediated signaling events [6]. Within this framework, GSH supplementation can be viewed as a targeted approach to reestablish intracellular thiol homeostasis while preserving physiological ROS functions, aligning with the concept of controlled redox equilibrium rather than universal antioxidant suppression.

### 3.2. Glutathione Deficiency in T2DM

Hyperglycemia itself depresses intracellular cysteine and total GSH in immune cells, furnishing direct evidence for a precursor bottleneck in diabetes. In monocytes from people with diabetes and in complementary animal models, elevated glucose levels reduced L-cysteine and GSH, rendering cells more susceptible to oxidative injury [7]. This depletion impairs thiol-dependent enzyme systems and can diminish innate immune function. The finding is crucial because it operationalizes the link between glucose toxicity and substrate scarcity for GSH synthesis. Therapeutically, N-acetylcysteine (NAC) or combined precursor strategies should replenish cysteine pools and restore GSH synthesis rates. This mechanism is compatible with observed improvements in oxidative stress markers in clinical settings and provides a rationale for pairing glycemic control with precursor supplementation in redox deficits.

Together, these data define glutathione deficiency as a convergent mechanism linking oxidative stress, immune dysfunction, and metabolic impairment in T2DM [1,2,3,4,5,6,7].

A 2024 analysis emphasized the interconnected roles of GSH deficiency, mitochondrial dysfunction, and insulin resistance in diabetes, proposing that targeted supplementation could ameliorate multiple pathophysiological pathways simultaneously [8]. The review underscored how GSH insufficiency contributes not only to glycemic dysregulation but also to systemic inflammation and endothelial dysfunction. By highlighting these intersections, the review framed GSH as a nodal point in diabetic pathophysiology and a promising therapeutic target.

A double-blind trial of individuals with obesity, both with and without T2DM, found that oral GSH supplementation improved insulin sensitivity, implying that GSH’s effects may extend beyond simple ROS scavenging. This is possibly through regulation of insulin receptor phosphorylation, enhancement of mitochondrial oxidative efficiency, or modulation of glucose transporter activity. These results highlight the multifaceted functions of GSH, which include metabolic regulation in addition to its role in oxidative stress buffering [9]. Despite showing promise within insulin sensitivity, the trial did not alter markers of oxidative stress, displaying discrepancies between this trial and others. This trial would ideally be conducted in a larger trial without such a limited timeframe of 3 weeks. Overall, the trial evidence positions GSH supplementation as a plausible adjunctive therapy for restoring insulin sensitivity and metabolic balance in individuals with obesity and diabetes.

Likewise, lipid peroxidation remains a durable biochemical signature in poorly controlled T2DM, and its relationship with the GSH system is bidirectional. In an intervention study, patients with higher HbA1c exhibit elevated lipid peroxidation markers alongside altered GSH peroxidase (GPx) activity, underscoring an overwhelmed peroxide–detoxification axis [10]. These correlations situate the GSH–GPx pathway at the intersection of glycemic burden and oxidative injury to membranes and lipoproteins. As glycemic control worsens, substrate pressure for GPx rises, and inadequate GSH availability limits enzymatic flux, fostering accumulation of peroxidized lipids. Clinically, this translates into vascular vulnerability and cellular dysfunction that propagate insulin resistance through inflammatory signaling. By positioning GPx activity as a functional biomarker, the data provide a tractable readout for antioxidant interventions. Interventions that raise intracellular GSH could normalize GPx-mediated detoxification and reduce lipid peroxidation burden. Such reductions would be expected to improve endothelial signaling and potentially macrovascular risk profiles when sustained. This framework also supports combining redox-targeted strategies with lipid-lowering therapies to address both substrate availability and oxidative processing. In trial design, co-primary endpoints that include peroxidation markers may capture clinically meaningful redox improvements.

## 4. GSH Supplementation Delivery & Nutrition

Beyond host cell biochemistry, oral GSH appears to influence gut microbial ecology, which is increasingly recognized as a determinant of metabolic inflammation. In T2DM patients on standard diabetes treatments, long-term GSH supplementation shifted microbial composition toward taxa often associated with improved metabolic tone and reduced inflammatory signaling [11]. Increased alpha diversity suggested a more resilient ecosystem, a property linked in other contexts to favorable metabolic outcomes. These microbial changes provide a plausible conduit between the luminal redox environment and systemic metabolic phenotypes, including insulin resistance. The observation that a redox-active nutrient can modulate the microbiome while pharmacotherapy remains constant strengthens causal inference for the intervention. Mechanistically, GSH may affect redox-sensitive microbial enzymes, thiol-dependent stress responses, or epithelial barrier interactions that feed forward into host inflammation. Importantly, microbiome modulation by GSH complements intracellular antioxidant effects rather than replacing them. This dual-axis action—microbial and host—invites composite endpoints spanning metabolomics, inflammatory mediators, and glycemic variability in future trials. Future microbiome-focused trial designs could help explain inter-individual heterogeneity in glycemic response to GSH supplementation and clarify dose and duration thresholds necessary to achieve durable ecological shifts (Figure 2).

Nutritional delivery of cysteine via whey protein isolate offers a practical route to augment GSH defenses in T2DM. In a randomized, placebo-controlled clinical trial, cysteine-rich whey protein improved oxidative-stress parameters, indicating that dietary proteins can shift systemic redox balance [12]. Such outcomes demonstrate that food-based precursors can deliver clinically relevant antioxidant effects without complex dosing regimens. The intervention feasibility is further strengthened by its favorable tolerability and compatibility with diabetes medications. Mechanistically, high-quality whey provides bioavailable cysteine, supporting γ-glutamylcysteine formation and downstream GSH synthesis. The trial suggests additive or synergistic potential when combined with glucose-lowering therapy, especially in patients with residual oxidative burden. Because dietary adherence can be tracked, these strategies lend themselves to pragmatic trials and real-world implementation. Nutritional approaches may also improve nitrogen balance and muscle health, secondary benefits relevant to older adults with T2DM [12]. Embedding redox endpoints within nutrition trials could clarify dose–response relationships for cysteine intake and guide formulation choices for long-term maintenance of GSH status.

Keratin-derived protein supplements extend the cysteine-delivery concept while offering a distinct amino-acid profile that may target skeletal muscle. In humans with T2DM, keratin protein improved glucose clearance and favorably modified muscle redox signatures, consistent with enhanced insulin sensitivity [13]. The tissue-specific effects underscore skeletal muscle as a primary locus where GSH-dependent redox state intersects with glucose uptake. By improving muscle antioxidant capacity, keratin may facilitate insulin-stimulated translocation of glucose transporters and reduce redox-induced signaling noise. This complements systemic strategies by focusing on the organ that disposes of the majority of postprandial glucose. The intervention’s practicality and tolerability advocate for broader evaluation across BMI strata and activity levels. The study also illustrates that not all protein sources are equivalent in redox outcomes. Future comparisons between keratin, whey, and plant-based proteins could refine cysteine delivery strategies and help define optimal protein matrices for sustained GSH support.

Metabolic crossover experimentation provides insight into how NAC interacts with dietary patterning to influence glucose dynamics. In a study manipulating glycemic index along with an NAC arm, investigators evaluated effects on glucose variability and β-cell function, generating pharmacodynamic data relevant to day-to-day management [14]. Results illustrated both the potential and the limits of NAC as an adjunct, with mixed effects across endpoints. Importantly, tolerability remained favorable, supporting iterative testing in combination with nutrition strategies. Such designs mirror clinical practice, where antioxidants, diet, and medication intersect. The data argues for refining timing and dosing of NAC relative to meals to maximize metabolic benefits. Continuous glucose monitoring and mixed-meal tolerance testing could sharpen the detection of subtle redox–metabolic interactions. Incorporating oxidative and inflammatory panels alongside glycemic metrics would create richer mechanistic readouts and guide the personalization of redox-adjunctive care in T2DM.

## 5. NAC Supplementation for Diabetic Complications

Pharmacologic cysteine provision via NAC has shown promise in diabetic neuropathy, a complication tightly coupled to oxidative stress. In a randomized controlled comparison with pregabalin, NAC improved neuropathic pain scores and reduced oxidative-stress biomarkers, indicating both symptomatic and biochemical benefits [15]. The results position NAC as a mechanistically targeted adjunct that addresses upstream drivers of nerve injury rather than only downstream pain pathways. Reductions in oxidative burden suggest improved neuronal or endothelial redox tone within affected nerves. Clinically, this could translate into slower progression of small-fiber dysfunction if sustained. NAC’s safety profile supports its integration with existing neuropathy regimens. These findings are limited in that future trials should add objective neurophysiology and small-fiber density endpoints to biochemical markers. This study also raises questions about whether earlier NAC initiation may serve as a preventative supplement in high-risk patients with rising oxidative markers. Lastly, combining glycemic optimization with NAC may yield additive benefits in nerve preservation, better reflecting real-world multimodal care.

Independent evidence from an 8-week randomized, controlled study further supports NAC’s role in neuropathy care. Adjuvant NAC improved neuropathic pain and favorably shifted oxidative-stress biomarkers, reinforcing the link between thiol repletion and symptom amelioration [16]. The repeated observation across trials strengthens confidence that the effect is not idiosyncratic to a single cohort. Mechanistically, NAC may protect endoneurial microvessels and reduce oxidative injury to axonal membranes, thereby lowering ectopic firing and dysesthesia. Biomarker improvements provide pharmacodynamic evidence that NAC meaningfully augments GSH-dependent defenses in neural tissue. Safety signals were acceptable, supporting outpatient use and potential chronic administration. These data justify larger, longer trials powered for functional endpoints, including nerve conduction and quantitative sensory testing. Incorporating quality-of-life measures would capture patient-relevant benefits beyond numeric pain scales. Stratifying baseline oxidative stress could identify responders and refine dosing. Together, these studies position NAC as a rational, redox-targeted adjunct for diabetic neuropathy, but require larger studies with defined endpoints to ensure clinical benefit.

Formulation science offers another lever for translating glutathione therapy to diabetic complications such as nephropathy. Liposomal encapsulation of GSH improved bioavailability and renal targeting in preclinical models, reducing oxidative stress, and attenuating pathological changes in diabetic kidneys [17]. This approach addresses limitations of conventional oral GSH, which can be variably absorbed and rapidly metabolized. By delivering intact GSH to renal tissue, liposomes enhance the probability of local redox correction where injury accrues. The resulting histological and biochemical improvements implicate oxidative stress as a modifiable driver of nephropathy progression. Such delivery systems could be combined with standard nephroprotective agents to amplify benefits. If extended to clinical trials, this data would determine whether targeted GSH delivery can achieve clinically meaningful reductions in albuminuria or eGFR decline.

## 6. GlyNAC Supplementation in Elderly Adults

Elderly adults—who share similar redox and metabolic features with T2DM—also may benefit from GSH. Supplementation corrected intracellular GSH deficiency, reduced systemic oxidative stress, and improved insulin resistance, echoing themes seen in T2DM studies [18]. Endothelial function also improved, aligning redox restoration with vascular health, a key target in diabetes management. The benefits suggest that GlyNAC addresses conserved nodes of metabolic dysregulation that cut across aging and diabetes. These data support trial designs that include both older adults with and without T2DM to test generalizability. Safety and adherence profiles were encouraging, a practical consideration for long-term prevention or adjunct therapy. Mechanistically, dual-precursor provision remains the distinguishing feature that may explain consistency across domains. Translationally, these findings justify exploring GlyNAC in combination with standard antidiabetic agents and lifestyle interventions. Multi-omic endpoints are required to map how redox restoration propagated through metabolic networks can help identify early surrogate markers predictive of durable clinical benefit.

Evidence from older adults receiving GlyNAC underscores the safety of precursor supplementation. One randomized control study reported that GlyNAC corrected intracellular GSH deficiency, reduced oxidative stress, and improved mitochondrial function without adverse safety signals in older adults with metabolic syndrome [19]. Participants tolerated the supplementation well, with no significant gastrointestinal, hepatic, or renal complications. Importantly, the older adult cohort often had comorbidities and polypharmacy, making safety findings highly translatable to T2DM patients. The trial also demonstrated that precursor supplementation could be delivered chronically without cumulative toxicity. The safety within the trial and improvements in insulin sensitivity and vascular function provide reassurance that GlyNAC can be applied safely in populations with metabolic dysfunction. However, numerous additional studies are necessary to verify this outcome as their intervention period was relatively short with a small sample size.

In a pivotal randomized controlled trial conducted with Indian patients, daily oral GSH supplementation significantly raised erythrocyte and plasma glutathione levels in elderly diabetic patients. Notably, benefits were especially marked in patients over 55 years old, suggesting that age-related depletion of glutathione exacerbates redox imbalance and worsens glycemic control. It also improved HbA1c, reduced oxidative DNA damage indicated by 8-hydroxy-2′-deoxyguanosine (8-OHdG), and stabilized fasting insulin levels [1]. However, patients who started diabetic drugs alone also saw a minor increase in GSH levels. These findings demonstrate that glutathione supplementation has the potential to replenish antioxidant stores in Indian elderly patients with T2DM and improves HbA1c, but broader inclusive trials comparing adjunctive glutathione with different diabetic drug regimens and stratified population subsets are needed to ensure this study is translatable for all T2DM patients.

## 7. Safety and Tolerability of GSH Supplementation and Precursors

Broader reviews have consistently emphasized NAC’s benign safety profile across diverse human studies. Kelly’s review detailed how NAC is a safe antidote for cysteine and GSH deficiency, noting that adverse effects were rare and typically mild [20]. A more recent comprehensive review examined NAC’s pharmacokinetics and systemic impacts in humans. Importantly, NAC showed no hepatotoxicity and instead demonstrated hepatoprotective properties, particularly in drug-induced injury contexts. Renal safety was also strong, with no adverse effects in patients with preexisting kidney disease. No cumulative toxicity or organ-specific damage was identified with chronic use. Importantly, NAC’s mechanism as a thiol donor and cysteine precursor avoided the pitfalls of broad-spectrum antioxidants that can disrupt physiological ROS signaling. NAC was also noted for being safe in children and elderly populations; these groups are often excluded from early-phase trials. The absence of mutagenic or carcinogenic signals provided further reassurance [21]; however, one study has noted caution with lung cancer models, where there may be an increase in proliferation as a result of p53 inhibition or likewise a possible increase in pulmonary hypertension. Because of this, future trial designs should apply caution with lung disease and focus on efficacy endpoints in other targeted organs. Together, these insights frame NAC as a pharmacological agent that is potent in action but requires safety to be investigated in specific scenarios.

In a separate review outlining NAC’s actions in oxidative stress reduction, inflammation modulation, and mitochondrial stabilization, these outcomes were achieved without safety compromises [22]. The most serious adverse effects reported historically were anaphylactoid reactions to intravenous administration, but these were rare and preventable with controlled infusion [20,22]. Oral NAC, relevant for chronic supplementation, was associated with even fewer adverse events. The review argued that NAC is unusual in being both mechanistically versatile and clinically safe, a rare combination in pharmacology. Unfortunately, titration studies showed that increasing doses beyond physiologic requirements yielded diminishing benefits rather than increasing risks [22]. In a similar review, authors focused on tolerability at therapeutic and supra-therapeutic doses. Oral doses up to 2400 mg/day were consistently safe, while higher doses used in acute settings were well tolerated under supervision. No evidence suggested reproductive, mutagenic, or carcinogenic risks with long-term exposure. Long-standing clinical use in acetaminophen toxicity served as a benchmark, demonstrating safe delivery of gram-level doses without concern for organ damage. The review also highlighted NAC’s compatibility with diverse drug regimens and absence of tolerance, reinforcing its utility in polypharmacy populations. Together, these studies reinforce NAC’s appeal as a chronic adjunct for oxidative stress-linked diseases and reinforce NAC as a reliable and safe intervention. However, given the high prevalence of MASLD (Metabolic dysfunction-Associated Steatotic Liver Disease) in T2DM, exploratory endpoints such as elastography and liver histology could be incorporated into trials to assess whether NAC modifies steatosis or inflammation. In the current literature microbiome–liver interactions remain largely unexplored and could represent an important determinant of GSH therapy response.

Systematic evaluation further confirms NAC’s safety across controlled clinical trials. A meta-analysis showed that NAC improved oxidative and inflammatory biomarkers without increasing adverse event rates compared to placebo. Withdrawal due to intolerance was rare and serious adverse events were exceedingly uncommon [23]. Gastrointestinal side effects were again the most frequent but were mild. Importantly, safety findings were consistent across diverse populations and dosing strategies. Oral and intravenous routes both demonstrated favorable safety, supporting generalizability. The consistency of findings across trials strengthens the conclusion that NAC is safe for long-term use and distinguishes it from antioxidants with narrower therapeutic windows [23]. These results justify the inclusion of NAC in chronic care regimens and conclude that NAC supplementation can be scaled in public health strategies without major safety barriers. Since dosage requirements are not always made clear in the literature, there is much work that needs to be done to provide patients with the best dosage necessary for their specific diseases.

Safety has also been demonstrated with direct GSH supplementation. Richie and colleagues conducted a six-month randomized control trial of oral GSH supplementation in adults and found it to be safe and well tolerated [24]. No significant hematological, hepatic, or renal adverse effects were reported. Plasma GSH levels rose significantly, confirming biological availability despite earlier skepticism about oral absorption. Side effects were minor and did not necessitate discontinuation. Importantly, the trial confirmed that exogenous GSH does not suppress endogenous synthesis, avoiding concerns of feedback inhibition. Long-term administration was feasible and safe, expanding options beyond precursor-based strategies [25]. This trial provided rare longitudinal human data for direct GSH supplementation, complementing decades of preclinical safety work. These results provide confidence that both direct and indirect strategies can be safely applied.

Collectively, these repeated reviews reiterate NAC’s safety profile across various contexts. By emphasizing NAC’s benign safety profile, the review strengthened its case for broader use in oxidative stress-driven diseases like T2DM [20]. Standardized dosing of GSH and its precursors remains variable across clinical studies, reflecting differences in formulation, duration, and target populations. However, research is lacking in clinical trials. The two human trials assessing oral supplementation have converged on moderate daily doses that effectively raise intracellular GSH without adverse effects (Table 2), illustrating that oral reduced form of GSH at 500–1000 mg per day and GlyNAC at approximately 100 mg/kg per component per day for 24 weeks were the employed regimens in recent clinical investigations. More inclusive clinical trials using specific clinical outcomes to measure efficacy would help solidify its safety profile in humans and allow for better translational application. Another limitation included its small sample size with a majority of females and limited time points.

## 8. Future Directions

Despite such encouraging data, significant research gaps remain. Standardization of biomarkers, dose–response mapping, long-term outcomes, and larger trials are required to move from proof-of-concept to incorporating GSH supplementation into clinical guidelines. Efforts to enhance GSH availability in clinical practice have revealed multiple strategies that require more rigorous evaluation in the setting of T2DM. These approaches include precursor supplementation, pharmacologic activation of glutamate-cysteine ligase, and transcriptional regulation through NRF2 [1]. Furthermore, analytical methods are not standardized across studies, complicating comparison and interpretation of results. Bioavailability also varies by formulation, and new delivery systems such as liposomal glutathione and sustained-release precursors need direct head-to-head evaluation. At the same time, interindividual variability driven by diet, genetics, and microbiome composition highlights the need for adaptive trial designs that stratify patients according to baseline thiol status. Long-term registries and mechanistic endpoints, such as mitochondrial function and redox proteomics, should be integrated into future studies to ensure that interventions deliver sustained biological and clinical benefit [11].

Within T2DM, there remains a need for randomized controlled trials comparing precursor combinations such as GlyNAC with single-agent approaches [3]. Future studies should also examine interactions with standard T2DM therapies, including metformin, SGLT2 inhibitors, and GLP-1 receptor agonists, to ensure compatibility and additive benefit. Clinical outcomes must expand beyond HbA1c to include continuous glucose monitoring data, endothelial function, and lipid or protein oxidation markers. Microvascular complications such as retinopathy, neuropathy, and nephropathy require targeted substudies with specialized readouts, while microbiome and metabolomic analyses could illuminate host–microbe interactions that influence redox metabolism. Importantly, implementation science is needed to assess real-world feasibility, adherence, and cost-effectiveness, particularly in populations disproportionately affected by T2DM [3].

Implementation challenges extend beyond pharmacology to adherence and real-world deployment. Comparative trials of sustained-release NAC, GlyNAC, and cysteine-rich dietary proteins are needed to define practical advantages [22]. Cost-effectiveness must be integrated into trial design, particularly in resource-limited settings with high T2DM burden. Regulatory inconsistencies regarding whether NAC is classified as a supplement or drug continue to hinder widespread uptake and standardization. Finally, safety monitoring should expand to include patient-reported outcomes, since mild but persistent side effects can undermine long-term adherence. Hepatology provides another rich source of insight, as NAC has demonstrated survival benefit in acute liver failure outside the context of acetaminophen toxicity [26,27].

Overall, future pharmacovigilance should prioritize long-term safety in contexts of polypharmacy, pregnancy, and frailty, but the existing evidence base is solid [28]. Ensuring consistent product quality through pharmacopeial standards will be essential to reduce variability between studies and across regions. Cost-effectiveness and equitable access must remain at the forefront, as T2DM disproportionately affects populations with fewer healthcare resources. Safety should also be reframed beyond simple absence of toxicity: interventions must preserve beneficial ROS-dependent signaling while correcting pathological oxidative stress. Patient-reported outcomes are indispensable for identifying tolerability issues that determine adherence. With this robust foundation, the next phase of research can responsibly explore the efficacy of glutathione-centered interventions in T2DM.

## 9. Conclusions

GSH supplementation and precursor therapies represent a promising but underdeveloped frontier in the management of T2DM. Current evidence demonstrates consistent biochemical efficacy and favorable safety across diverse populations, yet translation into clinical practice remains limited by methodological gaps, heterogeneity of endpoints, and insufficient long-term data. The strongest insights point toward NAC and GlyNAC as feasible, well-tolerated strategies to correct GSH deficiency, restore mitochondrial function, and mitigate oxidative injury. However, advances in trial design, biomarker standardization, and mechanistic readouts are needed to establish reproducible efficacy and define patient subgroups most likely to benefit. Importantly, future studies must not only demonstrate biological plausibility but also address implementation: affordability, adherence, and integration with existing diabetes therapies. With a solid foundation of safety and mounting mechanistic rationale, clinical research can transition from exploratory studies to large, multicenter trials capable of guiding evidence-based recommendations. If rigorously developed, GSH-centered strategies may ultimately serve as valuable adjuncts to current treatment paradigms, addressing persistent gaps in oxidative stress and improving outcomes in one of the most challenging chronic diseases of our time.

## Figures and Tables

**Table 1:** GSH’s, Glycine’s, and NAC’s underlying mechanisms, potential benefits, and shortfalls in the treatment of T2DM.

Supplement	Mechanism of Action	Potential Benefits in T2DM	Limitations
GSH (Direct Glutathione)	Direct replenishment of the central low-molecular-weight thiol and cellular antioxidant.	Improves insulin sensitivity in individuals with obesity and T2DM. Operates via localized or compartment-specific redox effects. Promising adjunctive therapy to restore both redox and metabolic balance.	Does not show global biomarker shifts, suggesting effects may be localized. Dependent on underlying precursor bottlenecks.
NAC (N-acetylcysteine)	Supplies L-cysteine, which is the primary rate-limiting substrate for de novo GSH synthesis.	Replenishes cysteine pools depleted by chronic hyperglycemia. Restores intracellular GSH synthesis rates in immune cells such as monocytes. Pairs effectively with glycemic control to resolve redox deficits and protect against glucose toxicity.	Only replenishes cysteine, leaving room for potential glycine deficiencies.
GlyNAC (Glycine + NAC)	Dual-precursor strategy supplying both rate-limiting substrates (glycine and cysteine) for de novo GSH synthesis.	Corrects impaired glutathione synthesis caused by diabetic precursor scarcity. Ameliorates multiple convergent pathways: glutathione deficiency, mitochondrial dysfunction, and insulin resistance. Improves mitochondrial fuel oxidation. Lowers systemic markers of oxidative stress and therefore mitigates endothelial dysfunction and inflammation.	Practicality in routine care still requires further dose optimization and durability testing. Currently relies on modest sample sizes in clinical data, necessitating larger phase II–III trials.

**Table 2:** GSH, Glycine, and N-acetylcysteine dosage and duration in clinical trials.

Study (Year)	Formulation/Route	Daily Dose	Duration
Richie et al., 2015 [24]	Reduced glutathione (oral)	500 mg or 1000 mg per day	6 months (24 weeks)
Kumar et al., 2021 [18]	GlyNAC (glycine + N-acetylcysteine, oral)	100 mg/kg each component per day	24 weeks
Panahi et al., 2022 [19]	N-acetylcysteine (NAC)	1800 mg per day	12 weeks

## Data Availability

No new data were created or analyzed in this study. Data sharing is not applicable to this article.
